# Mass Oral Azithromycin for Childhood Mortality: Timing of Death After Distribution in the MORDOR Trial

**DOI:** 10.1093/cid/ciy973

**Published:** 2018-12-17

**Authors:** Travis C Porco, John Hart, Ahmed M Arzika, Jerusha Weaver, Khumbo Kalua, Zakayo Mrango, Sun Y Cotter, Nicole E Stoller, Kieran S O’Brien, Dionna M Fry, Benjamin Vanderschelden, Catherine E Oldenburg, Sheila K West, Robin L Bailey, Jeremy D Keenan, Thomas M Lietman, Macrolides Oraux pour Réduire les Décès avec un Oeil sur la Résistance (MORDOR) Study Group

**Affiliations:** 1 Francis I. Proctor Foundation, University of California, San Francisco; 2 Department of Ophthalmology, University of California, San Francisco; 3 Department of Epidemiology and Biostatistics, University of California, San Francisco; 4 Institute for Global Health Sciences, University of California, San Francisco; 5 London School of Hygiene and Tropical Medicine, United Kingdom; 6 The Carter Center, Niamey, Niger; 7 The Dana Center, Johns Hopkins University School of Medicine, Baltimore, Maryland; 8 Blantyre Institute for Community Outreach, Blantyre; 9 College of Medicine, University of Malawi, Blantyre; 10 National Institute for Medical Research, Dar es Salaam, Tanzania

**Keywords:** childhood mortality, azithromycin, sub-Saharan Africa

## Abstract

In a large community-randomized trial, biannual azithromycin distributions significantly reduced postneonatal childhood mortality in sub-Saharan African sites. Here, we present a prespecified secondary analysis showing that much of the protective effect was in the first 3 months postdistribution. Distributing more frequently than biannually could be considered if logistically feasible.

**Clinical Trials Registration**. NCT02047981.

Trachoma programs have distributed >700 million doses of single-dose oral azithromycin to eliminate the strains of chlamydia that cause the blinding disease [1]. Azithromycin may also have collateral benefits against a number of infectious diseases including malaria, diarrhea, and pneumonia [2–7]. Several studies have suggested that mass azithromycin may reduce childhood mortality [8, 9]. The MORDOR trial (Macrolides Oraux pour Réduire les Décès avec un Oeil sur la Résistance) found that azithromycin distributions significantly reduced postneonatal childhood mortality by 14% across sites in Malawi, Niger, and Tanzania [10].

The timing and frequency of distributions in MORDOR were determined by logistical reasons. Secondary analysis of MORDOR could reveal the duration of azithromycin’s protective effect and suggest a preferential season for distributions. Here, we compared the survival time posttreatment in the azithromycin- and placebo-treated communities. In those children who died, we compared the distribution of time of death posttreatment in the 2 arms, and separately the season of treatment and the season of death.

## METHODS

MORDOR was a community-randomized trial conducted in the Malawian district of Mangochi, the Nigerien districts of Boboye and Loga, and the Tanzanian district of Kilosa [10]. The 1533 randomization units were the health surveillance assistant area in Malawi, the *grappe* in Niger, and the hamlet in Tanzania. Communities with a population between 200 and 2000 inhabitants on the most recent census were eligible for enrollment. Enrollment was based on census information available prior to the study. Communities remained in the study even if the population size drifted out of this numerical range. Children aged 1–59 months who weighed at least 3800 g were eligible for azithromycin or placebo. Biannual distributions were performed over each district in a rolling fashion, over a 6-month prespecified time period (8 months for the initial census and distribution) [10]. Thus, treatments could be given any time during the year. The estimated time of death was collected during the subsequent census.

### Statistical Analysis

We compared survival between the treatment and control groups using the log-rank test. Unfortunately, this survival analysis, in essence, reiterates the main finding of a mortality difference between the 2 groups—finding an overall difference in mortality between placebo and treatment provides no information regarding potential differences in timing. Thus, in addition, we prespecified a comparison of the distribution of observed all-cause mortality between the groups using the Cramer–von Mises statistic. In essence, this examines timing differences controlling for the fact that the azithromycin group experienced less mortality.

We then examined whether the time of death showed evidence of an annual cycle by testing whether or not the magnitude of the first trigonometric moment was nonzero, and whether the seasonality of deaths was different in the 2 groups by a test of equality of first trigonometric moments. Finally, we compared the times of treatments of individuals who (later) died using tests of equality of first and second trigonometric moments on an intent-to-treat basis (see Supplementary Data for details). In all cases, we conducted permutation tests by randomization unit (to account for clustering). All computations were performed in the R statistical programming language, version 3.5 for MacIntosh (R Foundation for Statistical Computing, R package survival).

## RESULTS

Children in communities randomized to azithromycin had a lower mortality hazard than those in placebo-treated communities. The log-rank test revealed evidence of a difference between the intervention and control groups (*P* = .004). Stratifying by country, we found a relative hazard of 0.81 (95% confidence interval [CI], .75–.87) for Niger, 0.96 (95% CI, .84–1.14) for Malawi, and 0.996 (95% CI, .80–1.24) for Tanzania (Cox proportional hazard model). Kaplan-Meier curves for each country are given in Supplementary Figure 1. This analysis is similar to the primary analysis reported elsewhere [10].

**Figure 1.**
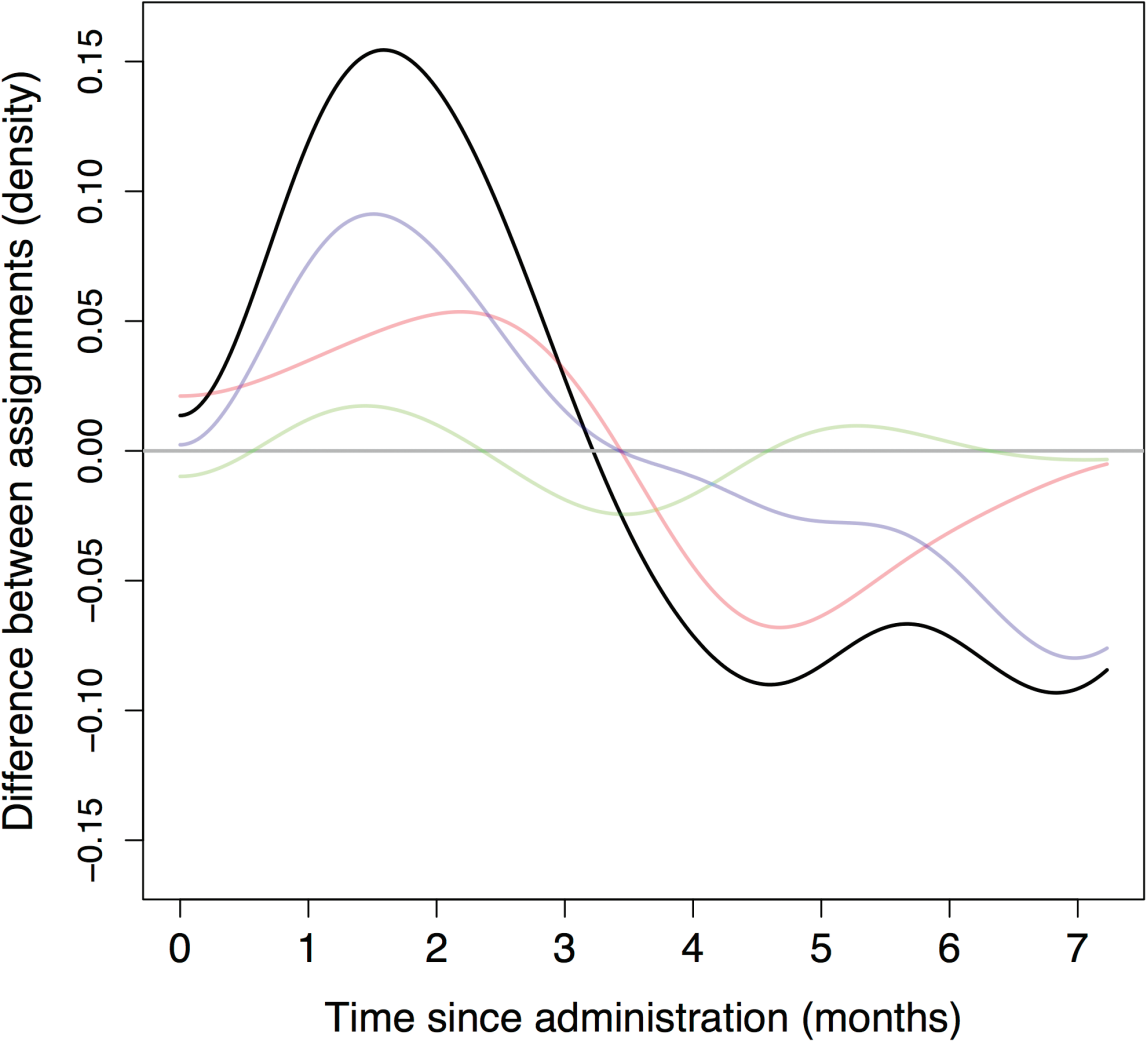
Excess mortality in the first 2 months in the control group, compared to the treatment group, is seen in the difference between estimated mortality time density estimates. The horizontal axis depicts time in days from the beginning of each phase. The vertical axis shows the difference between the density of mortality times in the control and treatment groups (smoothed with a Gaussian kernel of bandwidth 0.1 month). Results from Malawi are presented in red, Niger in purple, Tanzania in green, and total in black. Note that this displays the difference between 2 probability distributions, and thus an excess at one time would necessarily be balanced by a deficit at another.

It is possible that the timing of mortality among those who died may differ. Accordingly, for children who died, we compared the probability distribution of the time elapsed between mortality and drug administration between the placebo and azithromycin groups. The Cramer–von Mises test revealed evidence of a difference in the distribution of the time from drug distribution to death (*P* = .01). Overall in the 3 countries, among children who died, they were less likely to have died early in the treatment arm relative to the control arm (Figure 1). The black curve shows the difference in distribution of mortality times given that death occurred, adjusting for the fact that fewer deaths occurred in the treatment arm (note, in particular, that mortality in the treatment arm is lower than in the placebo arm, as shown by the Kaplan-Meier plots in the Supplementary Data). In addition, the difference in distribution of mortality times in each country is given in color, weighted by their relative contribution to the overall difference distribution.

We examined whether or not mortality reports occurred seasonally (Supplementary Figure 2). Specifically, we found evidence that the trigonometric mean occurrence time differed from zero, providing evidence of annual variation in mortality in Niger and Malawi (*P* < .001 and *P* < .001, respectively), but we did not find such evidence in Tanzania (*P* = .33). We next compared differences in seasonality between the 2 arms in each country on an annual scale (test of equality of first trigonometric moments) and on a twice-yearly scale (test of equality of second trigonometric moments) (see Supplementary Data for details). We found no evidence of a difference in the overall annual seasonal timing of deaths between the 2 arms on the annual scale in any country (*P* = .84, *P* = .78, and *P* = .33 for Niger, Malawi, and Tanzania, respectively). However, examination of the second trigonometric moment (corresponding to a twice-yearly frequency) suggests a change in seasonality (*P* = .02, *P* < .001, and *P* = .59 for Niger, Malawi, and Tanzania, respectively). The Supplementary Figures provide further information.

Similarly, treatments in all countries were not evenly distributed throughout the year: *P* < .001, *P* < .001, and *P* < .001 for Niger, Malawi, and Tanzania, respectively (Supplementary Figure 3). For those who died in the placebo arm, we found evidence that the treatments had been offered seasonally in Niger and Malawi (*P* < .001 and *P* < .001, respectively, test that the trigonometric mean equals zero), but not in Tanzania (*P* = .17). Finally, we compared the timing of placebo and azithromycin treatments for those who died (using the trigonometric mean). We found no evidence of a difference on the annual scale when comparing treatment times of those who died (*P* = .91, *P* = .84, and *P* = .93 for Niger, Malawi, and Tanzania, respectively).

## DISCUSSION

In the MORDOR trial, the hazard for death in children aged 1–59 months in the time after distribution was significantly lower in communities randomized to azithromycin compared to placebo. This confirmation of the trial results is not surprising as the analysis was similar in principle to the primary outcome where mortality was reduced 14%, estimated by negative binomial regression. However, we found that the distribution of the time from drug distribution to death differed significantly between the 2 arms. Note that this result, conditioned on a child having died, was not similar to the primary outcome. A general decrease in death could easily have resulted in a significant primary outcome, but not a different distribution contingent on death. That the largest effect was seen soon after the distribution is further evidence for an effect of oral azithromycin distributions. The deficit in deaths occurred in the first 3 months post–antibiotic distribution. This suggests that further protection might be achieved if distributions were given more frequently than biannually. If considered feasible, this would need to be evaluated in further trials.

The primary outcome of MORDOR was all-cause mortality, and analyses have so far not proven how exactly azithromycin had its effect [10]. Although the drug has modest anti-inflammatory properties, the high-dose protocol we followed is expected to yield a therapeutic antimicrobial concentration for approximately 1 week [11]. Experts thought that the most likely mechanism would be an effect on respiratory infection, diarrhea, or malaria [12]. If the major effect were against *Plasmodium falciparum*, we might expect treatments given near the malaria season (August–September) [13] to have a great effect on mortality. Evidence has suggested that childhood mortality risk in the Sahel (including Niger) is greatest when food supplies are scarce (the “lean season,” approximately May–September) [14] and during the malaria season [15], but we were unable to demonstrate that the season of death was different between the azithromycin- and placebo-treated communities. Similarly, we were unable to demonstrate that the season of treatment was an effect modifier, suggesting that any potential programs may not gain by timing distributions.

This secondary analysis has several limitations. For logistical reasons, the biannual MORDOR distributions were spread over 6-month time periods. Study teams were instructed to treat subdistricts in the same approximate order each period, with a gap from the previous treatment of between 4 and 8 months [10]. The flexibility in distribution time was thus logistical, not random, and perhaps not the most efficient for assessing the optimal timing of treatment. The large simple trial design precluded intensive data collection on individual children. As mortality was a relatively rare event, the power to assess whether timing of treatment was an effect modifier was low [16].

The previous MORDOR primary outcome proved that oral azithromycin distributions decrease all-cause mortality in postneonatal children in 3 sites in sub-Saharan Africa [10]. The distributions in the study were spread throughout the year, allowing assessment of any seasonal effects. Here, we were unable to demonstrate that the season of distribution was an effect modifier, so we cannot recommend specific timing for treatments. We were able to demonstrate that the greatest protection was found in the first 3 months postdistribution. Where feasible, quarterly distributions could be assessed.

## Supplementary Data

Supplementary materials are available at *Clinical Infectious Diseases* online. Consisting of data provided by the authors to benefit the reader, the posted materials are not copyedited and are the sole responsibility of the authors, so questions or comments should be addressed to the corresponding author.

ciy973_suppl_supplementary-Material

## Notes

***Financial support.*** The Bill & Melinda Gates Foundation provided the funding for the trial (grant number OP1032340). Pfizer Inc (New York City) provided both the azithromycin and the placebo oral suspensions. The Salesforce Foundation provided user licenses to Salesforce.com and cloud storage.

***Potential conflicts of interest.*** All authors: No reported conflicts. All authors have submitted the ICMJE Form for Disclosure of Potential Conflicts of Interest. Conflicts that the editors consider relevant to the content of the manuscript have been disclosed.
