# Supplementary material for: Mass Oral Azithromycin for Childhood Mortality: Timing of Death After Distribution in the MORDOR Trial
Source: Clin Infect Dis. 2018 Dec 17;68(12):2114–6. doi: 10.1093/cid/ciy973 (PMC6541729; doi:10.1093/cid/ciy973)
Supplement: ciy973_suppl_supplementary-Material [file ciy973_suppl_supplementary-material.docx]

**MORDOR Study Group**

The following investigators participated in MORDOR: *University of California, San Francisco, San Francisco, CA, USA – Catherine A Cook, Sun Y Cotter, Thuy Doan, Dionna M Fry, Bruce D Gaynor, Jeremy D Keenan, Elodie Lebas, Thomas M Lietman, Kieran S O’Brien, Catherine E Oldenburg, Travis C Porco, Kathryn J Ray, Philip J Rosenthal, George W Rutherford, Nicole E Stoller, Benjamin Vanderschelden, John P Whitcher, Zhaoxia Zhou, Lina Zhong; London School of Hygiene and Tropical Medicine, London, UK – Robin L Bailey, Sarah E Burr, John Hart, David CW Mabey, Anthony W Solomon; Johns Hopkins University, Baltimore, MD, USA – Evan M Bloch, Christian L Coles, Kurt Dreger, Hemjot Kaur, Alain B Labrique, Beatriz Munoz, Alfred Sommer, Jerusha Weaver, Sheila K West; Blantyre Institute for Community Ophthalmology, Blantyre, Malawi – Alvin Chisambi, Khumbo Kalua, Zachariah Kamwendo; University of Malawi College of Medicine, Blantyre, Malawi – Ken Maleta; The Carter Center, Atlanta, GA, USA – E Kelly Callahan, Aisha E Stewart; The Carter Center Niger, Niamey, Niger – Ahmed M Arzika, Abdou Moumouni Goundara, Salissou Kane, Ramatou Maliki; Programme National de Santé Oculaire, Niamey, Niger – Amza Abdou, Nassirou Beido, Boubacar Kadri; Muhimbili University of Health and Allied Sciences, Dar es Salaam, Tanzania – Mabula Kasubi; National Institute for Medical Research, Dar es Salaam, Tanzania – Leonard Mboera, Zakayo Mrango;International Trachoma Initiative, Decatur, GA, USA- Paul M Emerson.*

The steering committee for the trial consisted of the following investigators: Robin L Bailey, Jeremy D Keenan, Thomas M Lietman (PI), Travis C Porco, and Sheila K West.

**Acknowledgements**

We thank the program officers from the trial’s sponsor: *Bill & Melinda Gates Foundation,Seattle, WA, USA* – Rasa Izadnegahdar, Julie Jacobson, Thomas Kanyok, Erin Shutes. We also thank the members of the Data and Safety Monitoring Committee: *University of Washington, Seattle, WA, USA* – Judd L Walson; *Liverpool School of Tropical Medicine, Liverpool, UK* – Allen W Hightower; *Loyola University, Chicago, IL, USA* – Emily E Anderson, *Berhan Public Health & Eye Care Consultancy, Addis Ababa, Ethiopia* – Wondu Alemayehu; *Tulane University, New Orleans, LA, USA* – Latha Rajan.

**Statistical considerations**

*Survival analysis* was conducted using the logrank test (survdiff command, survival package, R program), with relative hazards determined by proportional hazards regression (coxph command, survival package, R program).

The Cramer-von Mises test was conducted to compare timing of mortality, in effect controlling for the fact that the number of deaths differed between the groups. This test, in essence, compares the differences in daily mortality counts between hypothetical cohorts experiencing the same number of total deaths over the study period. Figure 1 (main text) plots the difference in the densities; when the curve is positive, the placebo group experienced more mortality than the azithromycin group—adjusting for the difference in the overall mortality rate.

Analyses of seasonality were conducted using conventional circular (trigonometric) statistics^1^, where the time during a year is considered as an angle varying from 0 degrees (0 radians) at the beginning of the year, to 180 degrees (π radians) at approximately July 1, and so forth. The first trigonometric moment of angular occurrence times *x_j_*, j=1,…,N is a complex-valued quantity given by

$$\bar{x}=\frac{1}{N}\sum_{j=1}^{N} \left( \cos\left( x_{j} \right)+i sin(x_{j}) \right)$$

Observations uniformly spaced around the year correspond to a trigonometric/circular mean (first trigonometric moment) of 0. A seasonal pattern with two equal peaks six months apart also yields a trigonometric mean (first trigonometric moment) of zero. Any seasonal occurrence in which events occuring at one time are exactly balanced by an equal number occuring six months later yields a trigonometric mean of zero. The trigonometric mean measures an imbalance over the course of the year. In the most extreme case where all events occur at a single instant, the magnitude of the trigonometric mean is 1 (the largest possible value).

A test of seasonality based on trigonometric/circular means assesses whether the magnitude $\left| \bar{x} \right|$ differs from 0 more than would be expected by chance alone (given, for example, a uniform occurrence over a year). A test of equality of trigonometric/circular means tests whether $\left| \bar{x}-\bar{y} \right|$, derived from two samples *x* and *y*, is greater than that which would be expected under a null hypothesis of no difference in seasonality between the groups. We conducted the tests of equality using a permutation test, based on randomization group.

As an additional unplanned secondary analysis, we compared the second trigonometric moments between groups:

$$x=\frac{1}{N}\sum_{j=1}^{N} \left( \cos\left( 2x_{j} \right)+i sin(2x_{j}) \right)$$

The second trigonometric moment is very similar to the first, except it is taken with respect to a six month cycle. If an event happens on Jan. 1 and on Jul. 1, the first trigonometric moment is zero, but the magnitude of the second trigonometric moment is 1 (the maximum possible value).

**Results**

Kaplan-Meier survival curves are shown for each country (Niger: Figure S1a, Malawi: Figure S1b, Tanzania: Figure S1c).

Figure S1a. **Kaplan-Meier survival curves**, MORDOR trial, Niger subgroup. Results are shown for the first 240 days following treatment, aggregated over all 4 phases.

Figure S1b. **Kaplan-Meier survival curves**, MORDOR trial, Malawi subgroup. Results are shown for the first 240 days following treatment, aggregated over all 4 phases.

Figure S1c. **Kaplan-Meier survival curves**, MORDOR trial, Tanzania subgroup. Results are shown for the first 240 days following treatment, aggregated over all 4 phases.

Three additional figures show the distribution of crude mortality rates and treatment times over the year (Niger: Figure S1a, Malawi: Figure S2b, Tanzania: Figure S2c). Crude mortality rates were derived by dividing the number of deaths at each day by the estimated number of persons enrolled that day, followed by Gaussian smoothing with a bandwidth of one month. Our results in Niger are consistent with higher mortality following a summer “lean season” and malaria season in the Sahel^2,3^. We note that the seasonal timing is different in Malawi, with studies reporting peak malaria in approximately April or May^4^. In central Tanzania, studies have suggested lower mortality rates during June—September^5^. For Niger and Malawi, we found evidence of a difference in shape of the curve for those treated with azithromycin compared to the placebo group (evidenced by a change in the second trigonometric moment). We report rates per one thousand person-years (indicated on circle labels). Circles of constant rate are not equally spaced, so that equal areas correspond to equal values. Mortality rates for placebo are shown in blue, and for azithromycin in light red. Each country is shown on a different scale. The total number of deaths is indicated by N.

Figure S2a. **Seasonal timing of mortality** for the MORDOR trial, Niger subgroup. Rates per one thousand person-years (indicated on circle labels). Circles of constant rate are not equally spaced, so that equal areas correspond to equal values.

Figure S2b. **Seasonal timing of mortality** for the MORDOR trial, Malawi subgroup. Rates per one thousand person-years (indicated on circle labels). Circles of constant rate are not equally spaced, so that equal areas correspond to equal values.

Figure S2c. **Seasonal timing of mortality** for the MORDOR trial, Tanzania subgroup. Rates per one thousand person-years (indicated on circle labels). Circles of constant rate are not equally spaced, so that equal areas correspond to equal values.

We also show the distribution of treatment timing in each country over the year (aggregating both years together), showing the smoothed number of treatments per day (normalized to equal area to allow comparison of treatment times between placebo and azithromycin; Niger: Figure S3a, Malawi: Figure S3b, Tanzania: Figure S3c). The circle labels show treatment intensity relative to uniform treatment (i.e., 2 represents treatment at twice the daily rate as would occur if treatment occurred uniformly around the year). The total number of treatments given is indicated by N.

Figure S3a. **Seasonal timing of treatment** for the MORDOR trial, Niger subgroup (all phases). The circle labels show treatment intensity relative to uniform treatment. The total number of treatments given is indicated by N.

Figure S3b. **Seasonal timing of treatment** for the MORDOR trial, Malawi subgroup (all phases). The circle labels show treatment intensity relative to uniform treatment. The total number of treatments given is indicated by N.

Figure S3c. **Seasonal timing of treatment** for the MORDOR trial, Tanzania subgroup (all phases). The circle labels show treatment intensity relative to uniform treatment. The total number of treatments given is indicated by N.

**Supplemental References**

1. Pewsey A, Neuhauser M, Ruxto GD. **Circular Statistics in R.** Oxford University Press, Oxford, 2014.
2. Sié A, Tapsoba C, Dah C, Ouermi L, Zabre P, Bärnighausen T, Arzika AM, Lebas E, Snyder BM, Moe C, Keenan JD, Oldenburg CE. Dietary diversity and nutritional status among children in rural Burkina Faso. *International Health* 10(3):157-162, 2018.
3. Becher H, Kynast-Wolf G, Sié A, Ndugwa R, Ramroth H, Kouyaté B, Müller O. Patterns of malaria: cause-specific and all-cause mortality in a malaria-endemic area of west Africa. *American Journal of Tropical Medicine and Hygiene* 78(1):106-113, 2008.
4. Vaahtera M, Kulmala T, Maleta K, Cullinan T, Salin ML, Ashorn P. Epidemiology and predictors of infant morbidity in rural Malawi. *Paediatric and Perinatal Epidemiology* 14(4):363-371, 2000.
5. Alba S, Nathan R, Schulze A, Mshinda H, Lengeler C. Child mortality patterns in rural Tanzania: an observational study on the impact of malaria control interventions. *International Journal of Epidemiology* 43(1):204-215, 2014.
